# Association of modifiable risk factors and IL-6, CRP, and adiponectin: Findings from the 1993 Birth Cohort, Southern Brazil

**DOI:** 10.1371/journal.pone.0216202

**Published:** 2019-05-09

**Authors:** Ana Maria Baptista Menezes, Paula Duarte Oliveira, Fernando César Wehrmeister, Maria Cecilia F. Assunção, Isabel O. Oliveira, Luciana Tovo-Rodrigues, Gustavo Dias Ferreira, Helen Gonçalves

**Affiliations:** 1 Federal University of Pelotas—Postgraduate Program in Epidemiology, Pelotas, Brazil; 2 Federal University of Pelotas—Department of Physiology and Pharmacology, Pelotas, Brazil; Montana State University, UNITED STATES

## Abstract

**Background:**

The literature on the relationship between lifestyle behaviors and inflammatory markers is scarce.

**Methods:**

A birth cohort was followed since birth up to 22 years in Southern Brazil. Interleukin-6 (IL-6), C-reactive protein (CRP) and adiponectin were measured in nonfasting blood samples drawn at 18 and 22 years of age. Exposures including smoking, alcohol intake, physical inactivity and obesity, were collected at 15, 18 and 22 years. Cross sectional analyses were based on the number of follow-up visits with these exposures and the association with IL-6, CRP and adiponectin at 22 years old. We also carried out a longitudinal Generalized Least Squares (GLS) random-effects analysis with outcomes at 18 and at 22 years old. All analyses were adjusted for several covariates.

**Results:**

The sample comprised 3,479 cohort members at 22 years. The presence of obesity at ≥ 2 follow-ups showed the highest mean values (SE) for IL-6 [2.45 (1.05)] and CRP [3.74 (1.11)] and the lowest mean value for adiponectin [8.60 (0.37)] (adjusted analyses, females) compared with other exposures; the highest mean of IL-6 [1.65 (1.05)] and CRP [1.78 (1.11)] and the lowest mean of adiponectin [9.98 (0.38)] were for the number of follow-ups with ≥2 exposures compared to those with no exposures at any follow-up (adjusted analyses, females). The longitudinal analysis showed an increase in obesity associated with IL-6 and CRP in both sexes and an inverse association with adiponectin in females; smoking (in males) was associated with IL-6 and CRP, harmful alcohol intake was associated with CRP in males, and increased in physical activity was inversely associated with CRP in men.

**Conclusion:**

We concluded that obesity is the main exposure positively associated with IL-6 and CRP and inversely associated with adiponectin (mainly in females). Smoking is also associated with these markers in the longitudinal analysis (in males).

## Introduction

Increased levels of inflammatory markers, such as interleukin-(IL)-6 and C-reactive protein (CRP), predict the onset of poor health outcomes, particularly cardiovascular diseases and mortality [1, 2]. While the mechanisms that lead to increased values of these inflammatory markers are not completely understood, some risk factors, such as smoking, obesity and others, may be involved in the regulation of pro-inflammatory cytokines [3]; although circulating levels of IL-6 and CRP are physiologically linked, it remains unclear whether these markers track with one another with respect to several risk factors in healthy subjects. IL-6 stimulates the synthesis of CRP in the liver, and both markers are among the most commonly used indicators of inflammation.

Regarding adiponectin, an anti-inflammatory adipokine, epidemiological evidence has shown conflicting results [4]. An increase of 1 mg/mL in adiponectin concentration has been associated with either a decreased or an increased risk for cardiovascular events in chronic kidney disease patients [5, 6]. More detailed studies have shown that fat quality, and not fat mass, drives adiponectin expression [7].

Using a birth cohort from Southern Brazil, we aimed to examine the association between modifiable risk factors with information available during adolescence and the beginning of adulthood (smoking, alcohol consumption, physical exercise and obesity) and markers of inflammation (IL-6, CRP and adiponectin) at early adulthood. We further sought to determine the longitudinal effect of these risk factors on inflammatory markers.

## Methods

All hospital births that occurred in the calendar year of 1993 in the city of Pelotas, Southern Brazil were assessed by daily visits to all maternity hospital [8]. Of the 5,265 live births in the city, 5,249 were enrolled in our birth cohort study. Subsamples of the cohort were followed up during childhood [9], and all cohort members were sought when they had reached the mean age of 11, 15, 18 and 22 years. All cohort time-lines and methodologies can be found in previous publications [8, 10]. For this study, all the participants who agreed to donate blood samples at 22 years of follow-up were included. Nonfasting blood samples were drawn by venipuncture using vacutainer tubes at the 18- and 22-year-old follow-up visit; samples were processed in the laboratory, stored at ultralow temperature freezers in the same place and registered in a central biorepository. IL-6 was measured by the Quantikine HS Human IL-6 immunoassay kit (R&D Systems, Inc.; Minneapolis, MN55413, USA), C-Reactive Protein (CRP) was measured by immunoturdimetric assay (Labtest Diagnóstica SA, Minas Gerais, Brazil) and adiponectin was assayed with the ELISA Quantikine Human Total Adiponectin Immunoassay kit (R&D Systems, Inc., Minneapolis, USA). At 18 years, adiponectin was measured in a random subsample (n = 275) due to financial limitations. Intra-assay and interassay coefficients of variation were, respectively, 4.10% and 13.6% for IL-6 and 9.1% and 13.2% for adiponectin. The interassay coefficient for CRP was 2.0%. The exclusion criteria for the blood samples were refusal to collect blood and pregnancy.

The main exposures or risk factors were collected at ages 15, 18 and 22 years as follows: current smoking (Yes/No) defined as >6 days of cigarette consumption in the last month (at age 15 years–confidential questionnaire) or at least one cigarette/week in the last month at age 18 and 22 years; habitual alcohol intake (Yes/No) as follows: >6 days with alcohol consumption in the last month (at 15 years—confidential questionnaire) or harmful alcohol intake according to the AUDIT score ≥ 8 points [11] (at 18 and 22 years); physical inactivity defined as <300 min/week (at 15 and 18 years) or <150 min/week (22 years) by the International Physical Activity Questionnaire (IPAC); and obesity classified according to Body Mass Index (BMI; kg/m^2^) as >2 z-score (at 15 years) or ≥30 kg/m^2^ (at 18 and 22 years) [12, 13].

Each risk factor (smoking, alcohol intake, physical inactivity, obesity) could be categorized as not being present at any of the follow-up visits at 15, 18 or 22 years (0), as being present at one of the follow-up visits (1), or as being present at two or more of the follow-up visits (2+); for example, a cohort member who was not a smoker at 15, nor at 18 or at 22 years old was classified as 0 (zero exposure); someone who was a smoker either at 15 or at 18 or at 22 years old was classified as being exposed once (1); someone who was a smoker at two or at three follow-up visits was categorized as two or more (2+).

We also generated the variable named “number of follow-up visits with 2+ risk factors” (any 2+ risk factors without specifying which one; it could be smoking, or alcohol intake, or physical inactivity, or obesity) with the following categories: none (0) for those who had not been exposed to 2+ risk factors at any follow-up visit; 1 for those who had been exposed to two risk factors at 1 follow-up visit (either at 15 or at 18 or at 22 y); and 2+ for those who had been exposed to two risk factors at two or more follow-up visits (at any age of the follow-up visits).

The other covariables were skin color (white, black, brown and others); schooling (complete years); asset index (in quintiles); common mental disorders (SDQ score—15 years/ SRQ-20 score 18 and 22 years) [14]; medical diagnosis of asthma; diastolic blood pressure, and glucose, triglycerides, low density lipoprotein (LDL) and high density lipoprotein (HDL) (the last four available only at the 18 and 22 follow-ups).

Interviewers underwent standardization testing prior to beginning field work and every two months after to determine the repeatability and validity of the weight and height measurements.

The sample characteristics were described using absolute and relative frequencies for categorical variables and means and SE for continuous variables. Crude and adjusted linear regressions for the covariables above were conducted and all analyses sex-stratified due to interactions between sex and exposures in the majority of the analyses, mainly BMI. Additionally, we performed the Generalized Least Squares (GLS) random-effects model using the 18 and 22 follow-ups information (only two follow-ups with outcome information available). This model fits a linear regression model according to the longitudinal nature of the data and we used random effects assuming that the four exposures had been determined randomly at each follow-up. Stata version 12.2 software (Stata Corp., College Station, TX, USA) was used for the analysis, and the command “xtreg” for the GLS model; due to the asymmetric distribution of the variables (IL-6 and CRP) we performed the linear regression in the logarithmic scale; the results were reported in pg/mL, mg/L and μg/mL for IL-6, CRP and adiponectin, respectively (means and SE after exponential of the logarithm result) or logarithmic scale for CRP and IL-6 for GLS regression coefficients. P-values <0.05 in the Wald test for linear tendency were considered statistically significant.

Approval from the Federal University of Pelotas Ethics Committee was obtained for all follow-ups; the protocol numbers were 158/07, 05/11 and 1.250.366 for the, 15, 18 and 22 year follow-ups, respectively. A signature was requested from the cohort participants or their caregivers for informed consent prior to participation.

## Results

**Fig 1:**
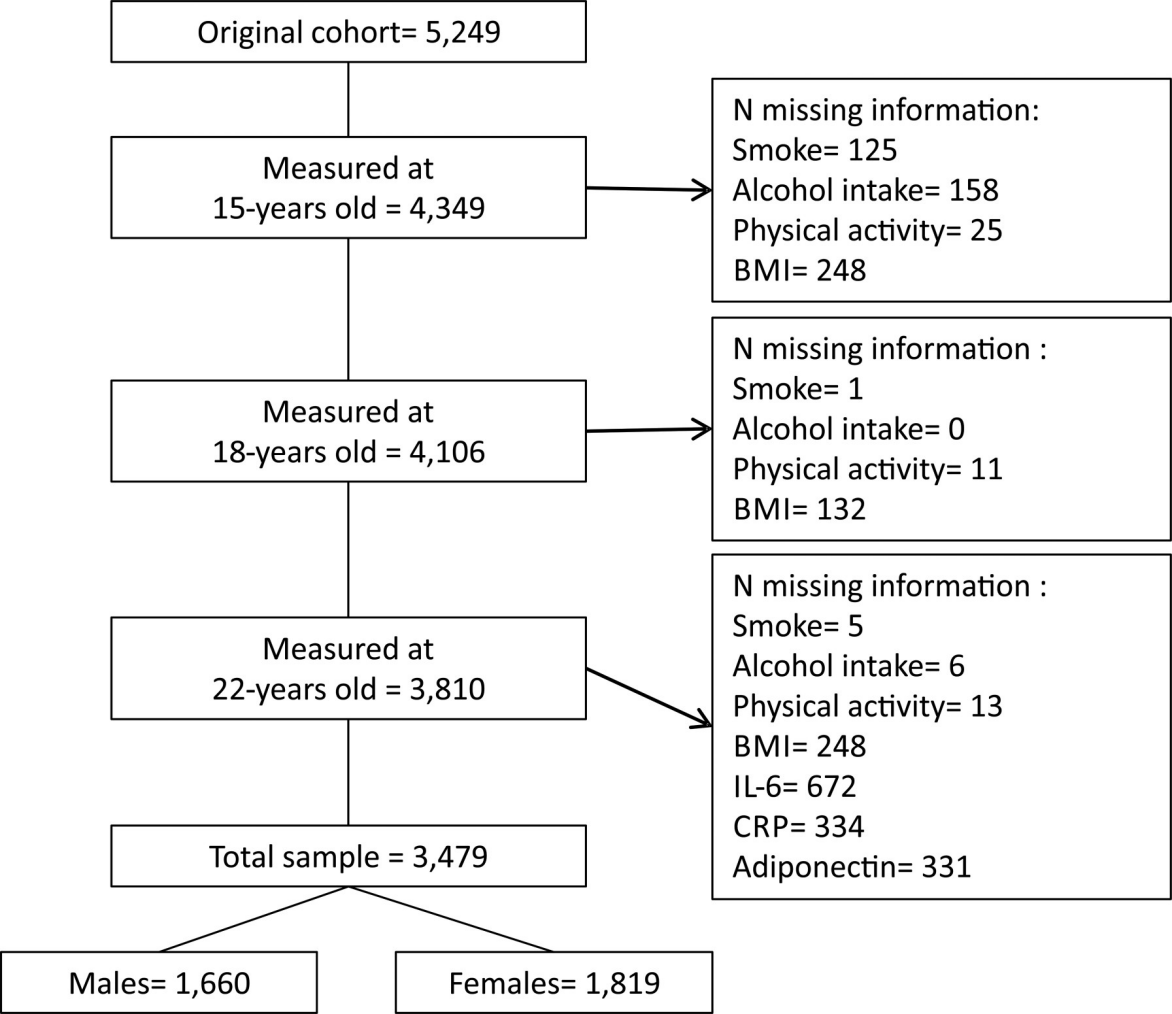
Sample flowchart. The 1993 Pelotas Birth Cohort.

The 22 year follow-up comprised 3,810 cohort participants who were located and agreed to be interviewed (Fig 1). However, we have missing information for some variables and not all participants donated blood (N = 331), comprising a total of 3,479 cohort members for the present analysis. From this total, 52.3% were females (Table 1), most of the subjects reported white skin color, 41.2% had 9–11 years of schooling and 22.6% of the men belonged to the richest asset index quintile compared to 17% of the women. A greater proportion of women had common mental disorders (SRQ-20) than men (23.6% versus 19.6%), and a medical diagnosis of asthma was slightly more prevalent among males (Table 1). In males, the total prevalence of smoking varied from 2.3% at 15 years to 20.7% at 22 years, being higher among males than females, except at age 15 years.

**Table 1:** Sample description (individuals with 22 years old interleukin-6, C-reactive protein and/or adiponectin information) according to demographic, socioeconomic and health variables, stratified by sex (n = 3,479). The 1993 Pelotas Birth Cohort.

	Males (n = 1,660) N (%)	Females (n = 1,819) N (%)
**Skin color**		
White	992 (63.6)	1,089 (62.7)
Black	232(14.9)	270(15.5)
Brown	267 (17.1)	320 (18.4)
Others	69 (4.4)	59 (3.4)
**Schooling (complete years)**		
0–4	70 (4.2)	28 (1.5)
5–8	544(32.9)	395 (21.7)
9–11	659 (39.8)	773 (42.5)
≥ 12	382 (23.1)	623(34.3)
**Asset index (quintiles)**		
1^st^	271 (16.3)	428 (23.6)
2^nd^	304 (18.3)	386 (21.2)
3^rd^	340 (20.5)	365 (20.1)
4^th^	369(22.2)	328 (18.1)
5^th^	375 (22.6)	310 (17.0)
**Common mental disorders (SRQ-20≥8 females and ≥6 males)**	323 (19.6)	427 (23.6)
**Medical diagnosis**		
Asthma	386 (23.3)	397 (21.8)
**Current Smoker**		
At 15 years	36 (2.3)	59 (3.4)
At 18 years	229 (14.6)	203 (12.0)
At 22 years	344 (20.7)	248 (13.6)
**Alcohol intake**		
At 15 years	69 (4.5)	81 (4.7)
At 18 years	552 (35.2)	286 (16.9)
At 22 years	501 (30.2)	260 (14.3)
**Physical inactivity**		
At 15 years	563 (36.1)	1,138 (65.5)
At 18 years	396 (25.3)	888 (52.7)
At 22 years	421 (25.4)	762 (42.0)
**Obese**		
At 15 years	147 (9.6)	117 (7.0)
At 18 years	128 (8.2)	174 (10.7)
At 22 years	221 (13.4)	338 (18.7)
	**Median (SD)**	**Median (SD)**
**Diastolic blood pressure (mmHg)**		
At 15 years	80.0 (10.2)	77.3 (9.4)
At 18 years	70.9 (7.8)	69.4 (7.7)
At 22 years	73.8 (8.6)	72.3 (8.7)
**LDL (mg/dL)**		
At 18 years	84.2 (20.0)	94.1 (23.7)
At 22 years	89.9 (24.2)	95.8 (25.3)
**HDL (mg/dL)**		
At 18 years	51.7 (8.8)	60.2 (10.9)
At 22 years	46.5 (10.8)	54.8 (13.3)
**Glucose (mg/dL)**		
At 18 years	93.7 (22.8)	89.0 (19.2)
At 22 years	91.7 (25.7)	88.5 (20.9)
	**Median (IQR)**	**Median (IQR)**
**Interleukin-6 (pg/mL)**	1.1 (0.8–1.7)	1.3 (0.9–2.0)
**C-reactive protein (mg/L)**	0.6 (0.3–1.4)	1.6 (0.6–4.1)
**Adiponectin (μg/mL)**	7.2 (5.1–10.0)	10.0 (7.3–13.5)
**Triglycerides (mg/dL)**		
At 18 years	68 (56–88)	70 (57–91)
At 22 years	85 (62–120)	87 (63–117)

Information based on 22-years follow-up, except where indicated.

SRQ-20: Self-Reporting Questionnaire; IQR = interquartile range

Obesity—BMI > 2 z-score (15 years) or ≥ 30kg/m^2^ (18 and 22 years)

Physical inactivity—< 300 min/week (15 and 18 years) or < 150 min/week (22 years)

Current smoker—> 6 days with cigarette consumption in the last month (15 years) or at least a cigarette /week in the last month (18 and 22 years)

Alcohol—current alcohol intake—> 6 days with alcohol consumption in the last month (15 years) or harmful alcohol intake—AUDIT score ≥ 8 points (18 and 22 years).

The same pattern was observed for alcohol consumption, i.e., a slightly higher prevalence of alcohol intake among females at 15 years and a higher prevalence among males at 18 and 22 years. Men were more active than women at all follow-ups, ranging from 63.9% to 74.6% at 15 and 22 years, respectively. Obesity showed the highest overall prevalence at 22 years (16.2%), being more prevalent among women than men at 18 and 22 years. Median diastolic blood pressure and glucose were higher among men and LDL, HDL and triglycerides were higher among women (at 18 and 22 years). The overall median (interquartile range) of IL-6, CRP and adiponectin was 1.2 pg/mL (IQR = 0.8–1.8), 1.0 mg/L (IQR = 0.4–2.7) and 8.6 μg/mL (IQR = 6.0–11.9), respectively; women showed higher values than men (Table 1).

**Fig 2:**
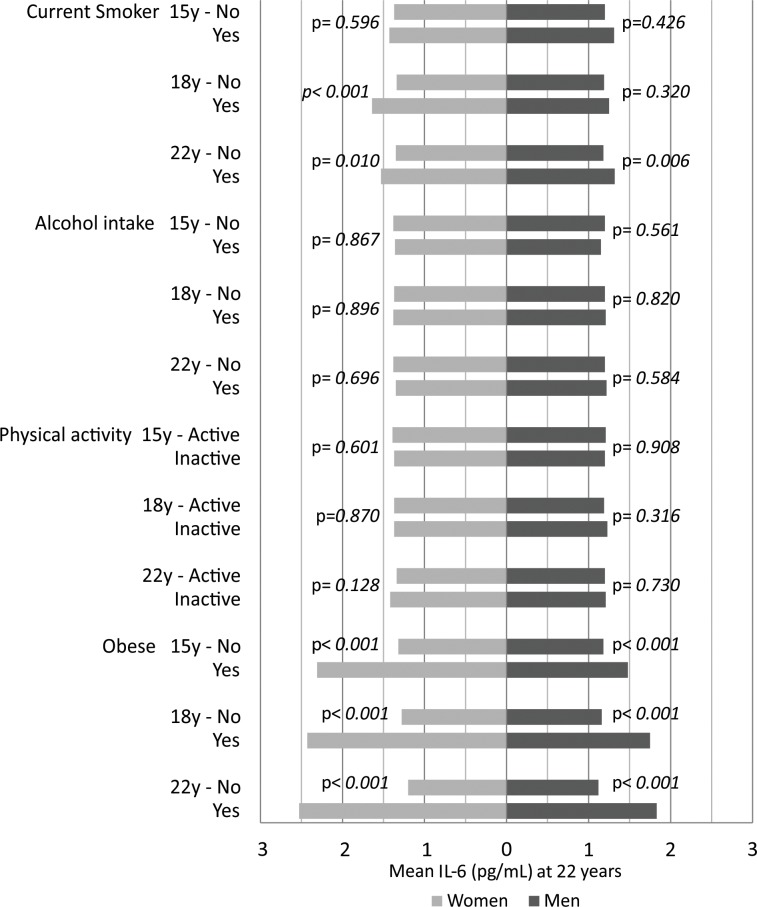
Mean levels of Interleukin-6 (IL-6) at 22 year follow-up according to smoking, alcohol intake, physical activity and obesity at 15, 18 and 22 years. The 1993 Pelotas Birth Cohort.

**Fig 3:**
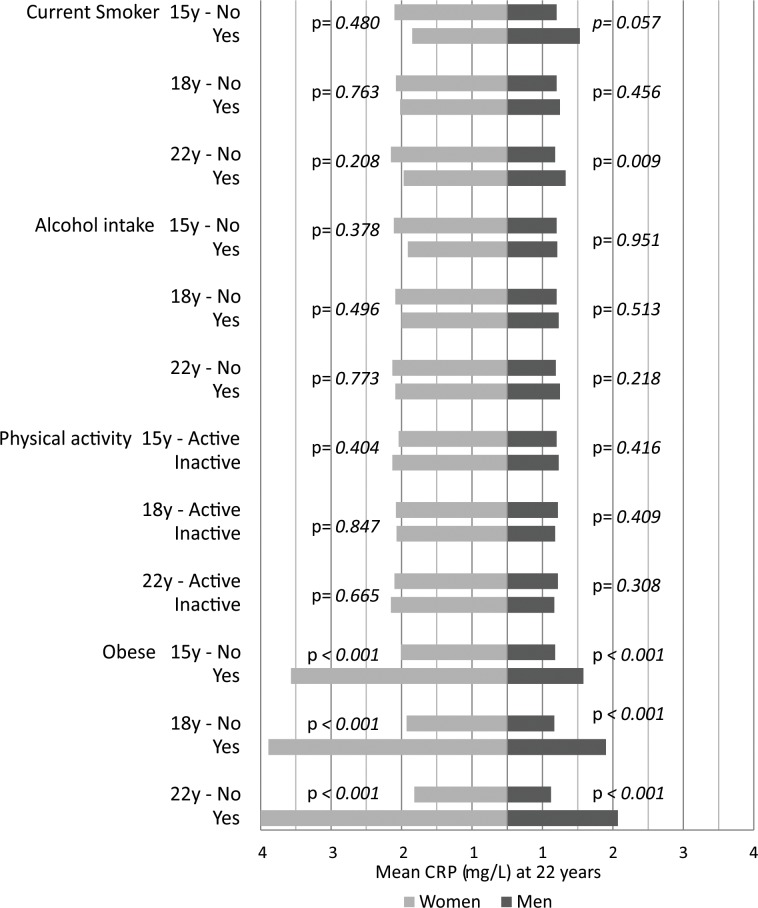
Mean levels of C-reactive protein at 22 year follow-up according to smoking, alcohol intake, physical activity and obesity at 15, 18 and 22 years. The 1993 Pelotas Birth Cohort.

**Fig 4:**
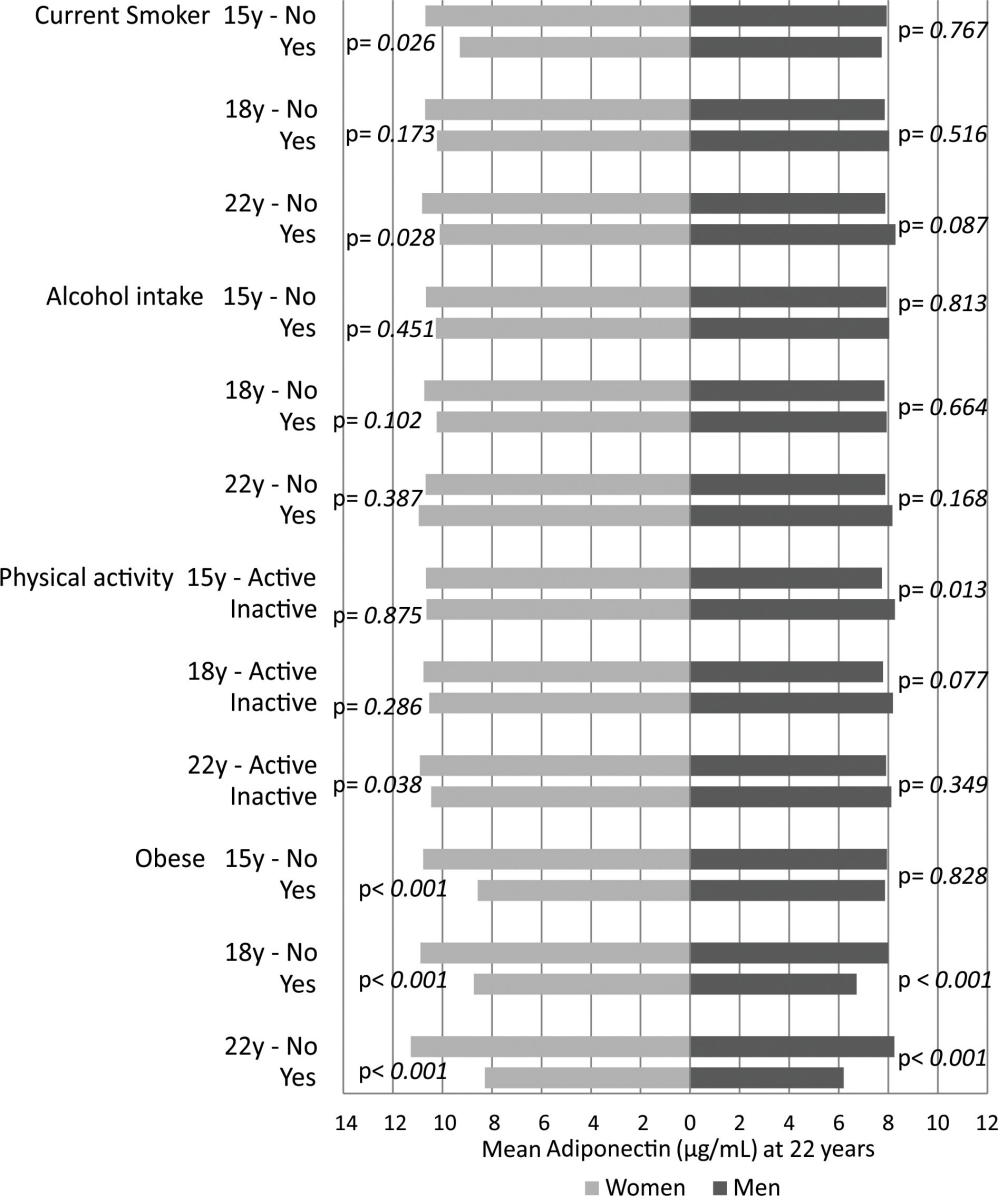
Mean levels of Adiponectin at 22 year follow-up according to smoking, alcohol intake, physical activity and obesity at 15, 18 and 22 years. The 1993 Pelotas Birth Cohort.

The majority of current smokers at 15, 18 and 22 years had higher values of IL-6 and CRP for both sexes, although a significant difference for IL-6 was only observed for men at 22 years and for women at 18 and at 22 years (Fig 2 and Fig 3). For CRP, statistical significance was present only for males at 22 years. Alcohol consumption and physical inactivity did not show statistical significance in the association with IL-6 and CRP (Fig 2 and Fig 3). Obesity was positively associated with IL-6 and CRP at all ages, in both sexes (p<0.001). Adiponectin was significantly lower in current smokers at 15 and at 22 years in females but not in men (Fig 4). No significant associations were observed for alcohol consumption or adiponectin in either sex at any follow-up. Inactive males showed a higher level of adiponectin compared with active males at 15 years, and inactive females showed a lower level at 22 years (Fig 4). Obesity was inversely associated with adiponectin in males at 18 and at 22 years and in females at all ages of follow-up (Fig 4).

In Tables 2 and 3, the crude and adjusted regressions are shown for the number of follow-ups (varying from 0 to ≥ 2 follow-ups) according to each exposure and the trajectory of the number of follow-ups with ≥ 2 risk factors from 15 to 22 years, for males and females, respectively. Smoking was associated with higher levels of IL-6 in the crude regression in both sexes, but significance was lost after adjustment for confounders. CRP was higher in male smokers only in the crude analysis but not in the adjusted analysis or in females. Although those exposed to alcohol intake in ≥ 2 follow-ups showed higher values of IL-6, there was no statistical significance in the crude or adjusted analysis. CRP was also higher for males exposed to alcohol in ≥ 2 follow-ups without statistical significance and was reduced according to the number of exposures in females. For subjects of either sex who were inactive in ≥ 2 follow-ups, IL-6 and CRP did not show any increases compared to those with no exposures. Adiponectin showed a direct association with being inactive in ≥ 2 follow-ups in males and females (Tables 2 and 3). There was a positive association for IL-6 and CRP in both sexes according to obesity (in the adjusted analysis); women who were obese in ≥ 2 follow-ups were associated with a mean IL-6 of 2.45 and a CRP of 3.74 compared to IL-6 of 1.21 and CRP of 1.29 for those with no exposures (adjusted analysis) (Table 3). Adiponectin showed an inverse association with obesity in ≥ 2 follow-ups in both sexes.

**Table 2:** Crude and adjusted linear regressions between number of follow-ups presenting each exposure, from 15 to 22 years, and levels of IL-6, CRP and adiponectin at 22 years old, males (n = 1,660). The 1993 Pelotas Birth Cohort.

	Prevalence N (%)	IL-6 (pg/mL) Mean (SE)		CRP (mg/L) Mean (SE)		Adiponectin (μg/mL) Mean (SE)	
		Crude	Adjusted	Crude	Adjusted	Crude	Adjusted
**Current smoker**		*p = 0*.*030*	*p = 0*.*235*	*p = 0*.*044*	*p = 0*.*783*	*p = 0*.*289*	*p = 0*.*197*
0	1,134 (76.7)	1.18 (1.06)	1.18 (1.02)	0.68 (1.04)	0.70 (1.04)	7.77 (0.12)	7.78 (0.12)
1	160 (10.8)	1.30 (1.02)	1.25 (1.06)	0.80(1.11)	0.71 (1.10)	8.08 (0.31)	8.03 (0.32)
2+	184 (12.5)	1.29 (1.05)	1.25 (1.05)	0.80 (1.09)	0.72 (1.10)	8.20 (0.29)	8.18 (0.31)
**Current /Harmful alcohol intake**		*p = 0*.*329*	*p = 0*.*623*	*p = 0*.*089*	*p = 0*.*303*	*p = 0*.*485*	*p = 0*.*153*
0	741 (50.6)	1.18 (1.03)	1.19 (1.02)	0.68 (1.04)	0.70 (1.05)	7.77 (0.14)	7.73 (0.15)
1	443 (30.2)	1.22 (1.03)	1.21 (1.03)	0.69 (1.06)	0.68 (1.06)	7.87 (0.19)	7.88 (0.19)
2+	281 (19.2)	1.22 (1.04)	1.21 (1.04)	0.80 (1.07)	0.78 (1.07)	8.10 (0.23)	8.14 (0.24)
**Inactive**		*p = 0*.*815*	*p = 0*.*996*	*p = 0*.*462*	*p = 0*.*234*	*p = 0*.*119*	*p = 0*.*047*
0	602 (40.4)	1.18 (1.03)	1.19 (1.03)	0.71 (1.05)	0.72 (1.05)	7.62 (0.16)	7.61 (0.16)
1	556 (37.3)	1.23 (1.03)	1.23 (1.03)	0.74 (1.05)	0.72 (1.05)	7.96 (0.17)	7.96 (0.17)
2+	332 (22.3)	1.18 (1.04)	1.18 (1.04)	0.66 (1.06)	0.65 (1.07)	8.14 (0.22)	8.13 (0.22)
**Obesity**		*p< 0*.*001*	*p< 0*.*001*	*p< 0*.*001*	*p< 0*.*001*	*p< 0*.*001*	*p = 0*.*001*
0	1,186 (81.7)	1.13 (1.02)	1.13 (1.02)	0.63 (1.03)	0.62 (1.03)	8.08 (0.11)	8.06 (0.12)
1	134 (9.2)	1.48 (1.07)	1.44 (1.06)	1.03 (1.11)	1.01 (1.11)	6.87 (0.34)	6.79 (0.35)
2+	131 (9.1)	1.69 (1.06)	1.68 (1.06)	1.47 (1.10)	1.43 (1.11)	7.15 (0.34)	7.21 (0.36)
**Number of follow-ups with 2+ risk factors**		*p< 0*.*001*	*p = 0*.*018*	*p< 0*.*001*	*p = 0*.*889*	*p = 0*.*283*	*p = 0*.*287*
0	918 (64.9)	1.13 (1.02)	1.16 (1.02)	0.64 (1.0)	0.69 (1.04)	7.79 (0.13)	7.74 (0.13)
1	313 (22.1)	1.27 (1.04)	1.24 (1.04)	0.78 (1.07)	0.72 (1.07)	8.16 (0.22)	8.19 (0.23)
2+	184 (13.0)	1.41 (1.05)	1.32 (1.05)	0.88 (1.08)	0.69 (1.10)	7.68 (0.29)	7.92 (0.32)

Regressions performed with Interleukin-6 (IL-6) and C-reactive protein (CRP) on logarithmic scale—results presented in exponential means. P-value by the Wald’s test for linear tendency. Adjusted for skin color, schooling (complete years), asset index (quintiles), common mental disorders (SDQ-20 score), diastolic blood pressure and medical diagnosis of asthma at 15 years-old + smoking (number of days smoking cigarettes in the last month), alcohol intake (number of days in the last month), physical activity (minutes per week) or BMI (z-score) according to exposure tested, at 15-years-old follow-up. Obesity—BMI > 2 z-score (15 years) or ≥ 30kg/m^2^ (18 and 22 years) Physical inactivity—< 300 min/week (15 and 18 years) or < 150 min/week (22 years). Current smoker—> 6 days with cigarette consumption in the last month (15 years) or at least a cigarette /week in the last month (18 and 22 years) Current alcohol intake—> 6 days with alcohol consumption in the last month (15 years) or harmful alcohol intake—AUDIT score ≥ 8 points (18 and 22 years).

**Table 3:** Crude and adjusted linear regressions between number of follow-ups, from 15 to 22 years, presenting the exposure and IL-6, CRP and adiponectin at 22 years old, females (n = 1,819). The 1993 Pelotas Birth Cohort.

	Prevalence N (%)	IL-6 (pg/mL) Mean (SE)		CRP (mg/L) Mean (SE)		Adiponectin (μg/mL) Mean (SE)	
		Crude	Adjusted	Crude	Adjusted	Crude	Adjusted
**Current smoker**		*p = 0*.*001*	*p = 0*.*242*	*p = 0*.*274*	*p = 0*.*120*	*p = 0*.*123*	*p = 0*.*879*
0	1,359 (82.3)	1.33 (1.02)	1.36 (1.02)	1.60 (1.04)	1.61 (1.04)	10.74 (0.13)	10.63 (0.13)
1	135 (8.2)	1.56 (1.06)	1.47 (1.06)	1.50 (1.13)	1.49 (1.12)	10.48 (0.40)	10.76 (0.40)
2+	158 (9.6)	1.57 (1.06)	1.42 (1.06)	1.43 (1.10)	1.35 (1.12)	9.96 (0.37)	10.65 (0.39)
**Current /Harmful alcohol intake**		*p = 0*.*931*	*p = 0*.*525*	*p = 0*.*329*	*p = 0*.*323*	*p = 0*.*632*	*p = 0*.*701*
0	1,191 (72.5)	1.36 (1.02)	1.37 (1.02)	1.61 (1.04)	1.61 (1.04)	10.71 (0.14)	10.66 (0.13)
1	326 (19.9)	1.45 (1.04)	1.42 (1.04)	1.50 (1.07)	1.47 (1.08)	10.52 (0.26)	10.68 (0.26)
2+	125 (7.6)	1.27 (1.06)	1.26 (1.06)	1.48 (1.12)	1.50 (1.12)	10.36 (0.42)	10.42 (0.42)
**Inactive**		*p = 0*.*441*	*0*.*074*	*p = 0*.*674*	*p = 0*.*304*	*p = 0*.*116*	*p = 0*.*006*
0	205 (12.3)	1.35 (1.05)	1.33 (1.05)	1.56 (1.09)	1.51 (1.10)	11.16 (0.32)	11.20 (0.32)
1	558 (33.6)	1.35 (1.03)	1.31 (1.03)	1.54 (1.06)	1.51 (1.06)	10.75 (0.20)	10.89 (0.20)
2+	898 (54.1)	1.39 (1.2)	1.41 (1.02)	1.60 (1.04)	1.63 (1.05)	10.45 (0.16)	10.36 (0.16)
**Obesity**		*p< 0*.*001*	*p< 0*.*001*	*p< 0*.*001*	*p< 0*.*001*	*p< 0*.*001*	*p< 0*.*001*
0	1,251 (80.0)	1.20 (1.02)	1.21 (1.02)	1.29 (1.11)	1.29 (1.04)	11.20 (0.13)	11.16 (0.13)
1	151 (9.7)	2.13 (1.06)	2.09 (1.05)	3.16 (1.04)	3.24 (1.11)	8.80 (0.37)	8.96 (0.37)
2+	163 (10.4)	2.57 (1.05)	2.45 (1.05)	3.71 (1.09)	3.74 (1.11)	8.37 (0.36)	8.60 (0.37)
**Number of follow-ups with 2+ risk factors**		*p< 0*.*001*	*p< 0*.*001*	*p< 0*.*001*	*p = 0*.*005*	*p< 0*.*001*	*p = 0*.*007*
0	1,025 (66.5)	1.22 (1.02)	1.27 (1.02)	1.38 (1.04)	1.44 (1.04)	11.15 (0.14)	10.95 (0.15)
1	330 (21.4)	1.63 (1.04)	1.56 (1.04)	2.00 (1.08)	1.91 (1.08)	10.05 (0.25)	10.29 (0.26)
2+	186 (12.1)	1.90 (1.05)	1.65 (1.05)	2.07 (1.10)	1.78 (1.11)	9.22 (0.34)	9.98 (0.38)

Regressions performed with Interleukin-6 (IL-6) and C-reactive protein (CRP) on logarithmic scale—results presented in exponential means. P-value by the Wald’s test for linear tendency. Adjusted for skin color, schooling (complete years), asset index (quintiles), common mental disorders (SDQ-20 score), diastolic blood pressure and medical diagnosis of asthma at 15 years-old + smoking (number of days smoking cigarettes in the last month), alcohol intake (number of days in the last month), physical activity (minutes per week) or BMI (z-score) according to exposure tested, at 15-years-old follow-up. Obesity—BMI > 2 z-score (15 years) or ≥ 30kg/m^2^ (18 and 22 years). Physical inactivity—< 300 min/week (15 and 18 years) or < 150 min/week (22 years). Current smoker—> 6 days with cigarette consumption in the last month (15 years) or at least a cigarette /week in the last month (18 and 22 years). Current alcohol intake—> 6 days with alcohol consumption in the last month (15 years) or harmful alcohol intake—AUDIT score ≥ 8 points (18 and 22 years).

The percentage of the number of follow-ups with ≥ 2 risk factors found in the category of ≥ 2 follow-up visits was similar between males and females (13% and 12.1%, respectively) (Tables 2 and 3). An association was found between the levels of IL-6 in both sexes (crude and adjusted analysis) and the number of follow-ups with 2 or more exposures in the period, i.e., the highest number of follow-ups and number of exposures was observed for IL-6; a similar pattern was observed for CRP but only for females (Table 3). The inverse association was observed for adiponectin but only in females (Table 3).

Table 4 shows the crude and adjusted regression coefficients provided by GLS random-effects analysis taking into account two moments of exposures and outcomes measures (18 and 22 years). The increase in obesity showed a direct effect on IL-6 (males 0.36, 95% CI 0.28, 0.43; females 0.58, 95% CI 0.51, 0.64) and CRP (males 0.76, 95% CI 0.62, 0.90; females 1.00, 95% CI 0.86, 1.13) and an inverse relationship with adiponectin in females (-2.43 95% CI -4.37, -0.50). In males, we also observed a direct relationship between changes in smoking and IL-6 and CRP. Changes in physical inactivity were negatively associated with CRP (-0.12, 95% CI -0.21, -0.02), but only in males.

**Table 4:** Crude and adjusted GLS random-effects models between risk factors and IL-6, CRP and adiponectin at 18 and 22 years.

	**Males**					
	**IL-6 (log pg/mL)** **β (95% CI)**		**CRP (log mg/L)** **β (95% CI)**		**Adiponectin (μg/mL)** **β (95% CI)**	
	**Crude**	**Adjusted**	**Crude**	**Adjusted**	**Crude**	**Adjusted**
**Current smoker**	*p< 0*.*001* 0.14 (0.07; 0.20)	*p = 0*.*017* 0.08 (0.01; 0.14)	*p< 0*.*001* 0.22 (0.10; 0.33)	*p = 0*.*012* 0.15 (0.03; 0.27)	*p = 0*.*221* -1.33 (-3.47; 0.80)	*p = 0*.*059* -2.01 (-4.10; 0.08)
**Harmful alcohol intake**	*p = 0*.*017* 0.06 (0.01; 0.11)	*p = 0*.*058* 0.05 (-0.002; 0.10)	p *= 0*.*001* 0.16 (0.07; 0.25)	*p = 0*.*003* 0.14 (0.05; 0.23)	*p = 0*.*237* -1.05 (-2.78; 0.69)	*p = 0*.*215* -1.03 (-2.66; 0.60)
**Physical inactivity**	*p = 0*.*545* 0.02 (-0.04; 0.07)	*p = 0*.*431* -0.02 (-0.07; 0.03)	*p = 0*.*232* -0.06 (-0.16; 0.04)	*p = 0*.*016* -0.12 (-0.21; -0.02)	*p = 0*.*993* 0.01 (-1.81; 1.83)	*p = 0*.*717* 0.31 (-1.37; 2.00)
**Obesity**	*p< 0*.*001* 0.43 (0.35; 0.50)	*p< 0*.*001* 0.36 (0.28; 0.43)	*p< 0*.*001* 0.89 (0.76; 1.03)	*p< 0*.*001* 0.76 (0.62; 0.90)	*p< 0*.*001* -3.43 (-5.04; -1.82)	*p = 0*.*186* -1.20 (-2.99; 0.57)
	**Females**					
	**IL-6 (log pg/mL)** **β (95% CI)**		**CRP (log mg/L)** **β (95% CI)**		**Adiponectin (μg/mL)** **β (95% CI)**	
	**Crude**	**Adjusted**	**Crude**	**Adjusted**	**Crude**	**Adjusted**
**Current smoker**	*p< 0*.*001* 0.15 (0.08; 0.22)	*p = 0*.*823* 0.01 (-0.06; 0.08)	*p = 0*.*636* 0.03 (-0.11; 0.18)	*p = 0*.*614* -0.04 (-0.18; 0.11)	*p = 0*.*424* -0.92 (-3.19; 1.33)	*p = 0*.*488* 0.89 (-1.63; 3.42)
**Harmful alcohol intake**	*p = 0*.*448* 0.03 (-0.04; 0.09)	*p = 0*.*451* 0.02 (-0.04; 0.09)	*p = 0*.*298* 0.07 (-0.06; 0.20)	*p = 0*.*202* 0.08 (-0.05; 0.21)	p = 0.968 0.05 (-2.21; 2.30)	*p = 0*.*784* -0.31 (-2.56; 1.93)
**Physical inactivity**	*p = 0*.*430* 0.02 (-0.03; 0.07)	*p = 0*.*681* 0.01 (-0.03; 0.05)	*p = 0*.*783* -0.01 (-0.11; 0.08)	*p = 0*.*973* -0.002 (-0.09; 0.09)	p = 0.587 0.51 (-1.33; 2.35)	*p = 0*.*729* 0.31 (-1.43; 2.04)
**Obesity**	*p< 0*.*001* 0.68 (0.62; 0.74)	*p< 0*.*001* 0.58 (0.51; 0.64)	*p< 0*.*001* 1.12 (0.99; 1.25)	*p< 0*.*001* 1.00 (0.86; 1.13)	*p< 0*.*001* -4.22 (-5.77; -2.67)	*p = 0*.*014* -2.43 (-4.37; -0.50)

Adiponectin at 18 years available in a subsample n = 259. Sample with IL-6 and CRP information at 18 and 22 years n = 2989.

Adjusted for skin color, schooling (complete years), asset index (quintiles), common mental disorders (SDQ-20 score), HDL, LDL, glucoses, triglycerides, diastolic blood pressure, asthma medical diagnosis. Current smoking (at least a cigarette /week in the last month), harmful alcohol intake (AUDIT score ≥ 8 points), physical inactivity (< 300 min/week at 18 years or < 150 min/week at 22 years) and obesity (BMI ≥ 30kg/m^2^), were added together in the model. All variables were collected at 18 and 22 year follow-ups, except skin color. P-value by the chi-squared Wald’s test

## Discussion

This study demonstrates a direct association between the number of follow-up visits exposed to risk factors, such as smoking, alcohol consumption, inactivity and obesity as well the number of the number of risk factors, and the levels of IL-6 and CRP and an inverse association with adiponectin in a young adult Birth Cohort in Southern Brazil.

The analysis of each exposure shows obesity as the main variable responsible for the association. The magnitude of the effect points out that obesity being present at two or more of the follow-up visits is associated with 48.7% and 110% of increase in IL-6 and CRP, respectively, and a decrease of 11% in adiponectin, in males; higher magnitude is observed among females, with 102% and 189% of increase in IL-6 and CRP, respectively, and a decrease of 23% in adiponectin (findings from the adjusted linear regression in Tables 2 and 3).

Alcohol consumption and inactivity, as risk factors per se, did not show an association with IL-6 or CRP for either sex. For smoking females at 15 and 22 years and inactive females at 22 years, there was a low prevalence of adiponectin. Cardiovascular diseases [15, 16], diabetes [17], mental diseases [18], cognitive impairment [19], among several other diseases, have inflammation as one of their main physio pathological mechanisms, with smoking [20], alcohol [21], sedentary lifestyle [22, 23] and obesity [15, 16] as the most well known risk factors underlying these chronic conditions. It is biologically “plausible” to think that the above lifestyle behaviors precede an inflammatory response and that inflammatory markers, such as IL-6, CRP, among others, can shed light on the pathways of several chronic inflammatory diseases. There is also evidence indicating IL-6 and CRP as predictors of mortality [24–26]. In contrast to most adipokines, adiponectin secretion—an anti-inflammatory marker—is downregulated in obese individuals [27, 28] and, according to some studies, can protect against mortality [29, 30]. Three independent meta-analyses did not find any evidence to suggest that lower levels of adiponectin are associated with an increased risk of cardiovascular disease or stroke [31–33].

A prospective population-based study in a nationally representative sample of elderly Costa Ricans found that women with a CRP level 2-SD above the mean had an 81% higher risk of dying in the coming year compared to women with a CRP level equal to the mean [25]. Sattar et al [26], in The Elderly at Risk Study, found that elevations in IL-6 levels were significantly more associated with fatal myocardial infarction or stroke death (hazard ratio 1.75 for 1 log unit increase in IL-6) than with risk of nonfatal cardiovascular death (hazard ratio 1.17), even after adjustments; similar trends were found for CRP.

Unfortunately, several studies on inflammatory markers and lifestyle exposures have been conducted through cross-sectional study designs; although exposures, such as smoking, alcohol, sedentary lifestyle and obesity, appear to precede inflammation, temporality cannot be ascertained in cross-sectional studies, and doubt regarding the direction of the association continues to be a pertinent limitation [34–36]. Most longitudinal studies in the literature on this field come from developed countries [37, 38], and cohort studies evaluating IL-6, CRP and adiponectin in Latin America countries, specifically at early adulthood, are scarce [39–41].

A study conducted among Puerto Rican adults aged 45–75 years living in Boston [35] found a significant and inverse association between a validated Healthy Lifestyle Score (HLS), which included adherence to five behavioral components (diet, physical activity, smoking, social support and sleep), and IL-6 and TNF-alpha (TNF-α). For each 20-unit increase in the HLS, there was a decrease in IL-6 (β ± SE = −0.55 ± 0.13; *P*<0.001) and TNF-α (−0.39 ± 0.13; *P* = 0.004) levels; the same did not happen with CRP after adjustment.

In the 1982 Pelotas Birth Cohort carried out in the same city in Brazil as the present 1993 Cohort, the authors assessed the association between CRP and obesity, smoking and alcohol, among other variables in a cross-sectional approach; a direct association was found between CRP and obesity, smoking (only in males), and no association with alcohol consumption [42]. IL-6 was not available for the 1982 cohort, and the authors did not assess the cumulative effect of risk factors. In a longitudinal analysis of the 1982 Pelotas Cohort, the effect of life course socioeconomic indicators was evaluated on CRP at age 23 years, and adiposity accounted for the overwhelming majority of the associations between socioeconomic status and CRP levels [41].

Most findings in the literature are consistent regarding the association of obesity and higher levels of IL-6 and CRP, corroborating our results. For smoking, several authors have also found an association with inflammatory biomarkers [43, 44], but this has not been as consistent as for obesity [45, 46].

Risk factors, such as alcohol intake and level of physical activity, have shown controversial results in the literature, varying from direct associations, lack of association, mainly after adjusting for confounders, to inverse associations, i.e., between physical activity and inflammatory biomarkers [47–49]. In a cohort of approximately 2,500 older subjects for both sexes in the US, alcohol intake showed a J-shaped relationship with IL-6 and CRP levels, i.e., those who never drank compared with those who drank 8 or more drinks per week had an increased likelihood of high levels of both IL-6 and CRP compared with subjects who consumed 1 to 7 drinks per week [48]. Possible explanations for these findings, since they have also been shown in other studies [50–52], are related to the involvement of inflammatory cytokines in the early stage of many liver diseases, supporting the association of heavy alcohol intake and increased levels of IL-6 and CRP. Nevertheless, there is experimental evidence that alcohol intake enhances the metabolism of IL-6, but not TNF-α [53], and might downregulate IL-6 and TNF-α secretion from adipocytes [54]; these are some proposed beneficial effects of ethanol on IL-6. Our study evaluated alcohol consumption in two different ways; at 15 years, data was obtained regarding current habitual alcohol intake defined by the time of more than 6 days consuming alcohol in the last month, and at 18 and 22 years, the AUDIT tool was used as a score for the definition of harmful alcohol intake. No clear pattern was observed in our study for CRP or IL-6 levels and alcohol consumption at the three follow-ups in both sexes; even when analyzing cumulative follow-ups of alcohol consumption adjusted for confounders, there was an increase in the mean levels of IL-6, CRP and adiponectin in men and a decrease of all markers in females (without statistical significance).

Some cross-sectional papers have indicated that higher levels of physical activity are associated with lower levels of CRP [55–58] and IL-6 [56–58]; however, significant relationships between physical activity and markers of inflammation are not consistently reported [59, 60]. In the Multi-Ethnic Study of Atherosclerosis, a cohort of middle-aged to older adults in the USA, there is evidence that cumulative moderate-to-vigorous physical activity (regardless of intentionality) contributes to lowering inflammation [61].

In a clinical trial of healthy women from the USA, the authors found increased circulating levels of IL-6 and CRP with an increasing number of clinical cardiovascular risk factors (age > 60 year, current smoking, sedentary lifestyle, BMI > 27, systolic blood pressure ≥ 140 mmHg and diabetes); this was the only study found in the literature assessing the additive effects of several risk factors and IL-6 and CRP.

Some limitations in the present paper should be highlighted: a) the self-reported exposure to smoking and alcohol consumption, which may be prone to underreporting, and the differences between the data collection at 15 years (confidential questionnaire) and 18 and 22 years; b) the absence of other inflammatory markers, such as fibrinogen, TNF-α, leptin, and others; c) the possibility of residual confounding because smoking, alcohol consumption and physical inactivity are associated with other lifestyle characteristics; and d) and d) The lack of cytokines information in more than two time-points and adiponectin availability in a subsample at 18 years.

However, there are several strengths to be mentioned. There is a paucity of data in the literature evaluating risk factors with inflammatory markers in healthy individuals at early adulthood. Longitudinal studies, mainly in Latin America, are very scarce, and the high rates of follow-up in the present birth cohort and the assessment of several confounders reinforce our findings. Importantly, the data for obtaining the BMI measurements were of high quality.

## Conclusions

The association of inflammatory and anti-inflammatory markers with lifestyle modifiable risk factors and the additive effect of these risk factors in a healthy young population, analyzed in the present paper, can be of value in the prevention of chronic diseases.

## Supporting information

S1 File Analysis dataset (Stata v. 12). (DTA)

S1 Table Prevalence of risk factors according to age and mean (SE) levels of IL-6, CRP and adiponectin at 22 years, males. The 1993 Pelotas Birth Cohort.
(DOCX)

S2 Table Prevalence of risk factors according to age and mean (SE) levels of IL-6, CRP and adiponectin at 22 years, females. The 1993 Pelotas Birth Cohort.
(DOCX)
