# Supplementary material for: Association of modifiable risk factors and IL-6, CRP, and adiponectin: Findings from the 1993 Birth Cohort, Southern Brazil
Source: PLoS One. 2019 May 9;14(5):e0216202. doi: 10.1371/journal.pone.0216202 (PMC6508856; doi:10.1371/journal.pone.0216202)
Supplement: S1 Table — The 1993 Pelotas Birth Cohort. (DOCX) [file pone.0216202.s002.docx]

S1 Table. Prevalence of risk factors according to age and mean (SE) levels of IL-6, CRP and adiponectin at 22 years, males. The 1993 Pelotas Birth Cohort.

|  | **Prevalence**  **N (%)** | **IL-6 (pg/mL)**  **Mean (SE)** | **CRP (mg/L)**  **Mean (SE)** | **Adiponectin (µg/mL)**  **Mean (SE)** |
| --- | --- | --- | --- | --- |
| **15 years** |  |  |  |  |
| **Current Smoker** |  | *p= 0.426* | *p= 0.057* | *p= 0.767* |
| No | 1,498 (97.7) | 1.20 (1.01) | 0.70 (1.03) | 7.93 (0.10) |
| Yes | 36 (2.3) | 1.31 (1.09) | 1.03 (1.19) | 7.73 (0.66) |
| **Habitual alcohol intake** |  | *p= 0.561* | *p= 0.951* | *p=0.813* |
| No | 1,453 (95.5) | 1.20 (1.02) | 0.70 (1.03) | 7.92 (0.10) |
| Yes | 69 (4.5) | 1.15 (1.08) | 0.71 (1.16) | 8.04 (0.48) |
| **Physical activity** |  | *p=0.908* | *p=0.416* | *p= 0.013* |
| Active | 997 (63.9) | 1.21 (1.02) | 0.70 (1.04) | 7.75 (0.13) |
| Inactive | 563 (36.1) | 1.20 (1.03) | 0.73 (1.05) | 8.27 (0.17) |
| **Obese** |  | p< 0.001 | p< 0.001 | *p= 0.828* |
| No | 1,379 (90.4) | 1.18 (1.02) | 0.68 (1.03) | 7.95 (0.11) |
| Yes | 147 (9.6) | 1.48 (1.06) | 1.08 (1.10) | 7.87 (0.33) |
| **18 years** |  |  |  |  |
| **Current Smoker** |  | *p= 0.320* | *p= 0.456* | *p= 0.516* |
| No | 1,338 (85.4) | 1.19 (1.02) | 0.70 (1.03) | 7.86 (0.11) |
| Yes | 229 (14.6) | 1.25 (1.04) | 0.75 (1.08) | 8.04 (0.26) |
| **Harmful alcohol intake** |  | *p= 0.820* | *p= 0.513* | *p= 0.664* |
| No | 1,016 (64.8) | 1.20 (1.02) | 0.70 (1.04) | 7.85 (0.12) |
| Yes | 552 (35.2) | 1.21 (1.03) | 0.73 (1.05) | 7.94 (0.17) |
| **Physical activity** |  | *p= 0.316* | *p= 0.409* | *p= 0.077* |
| Active | 1,166 (74.7) | 1.19 (1.02) | 0.72 (1.04) | 7.79 (0.11) |
| Inactive | 396 (25.3) | 1.23 (1.03) | 0.68 (1.06) | 8.19(0.20) |
| **Obese** |  | *p< 0.001* | *p< 0.001* | *p< 0.001* |
| No | 1,425 (91.8) | 1.16 (1.02) | 0.67 (1.03) | 8.00 (0.10) |
| Yes | 128 (8.2) | 1.75 (1.06) | 1.40 (1.10) | 6.72 (0.35) |
| **22 years** |  |  |  |  |
| **Current Smoker** |  | *p= 0.006* | *p= 0.009* | *p= 0.087* |
| No | 1,316 (79.3) | 1.18 (1.02) | 0.68 (1.03) | 7.88 (0.11) |
| Yes | 344 (20.7) | 1.32 (1.04) | 0.83 (1.07) | 8.29 (0.21) |
| **Harmful alcohol intake** |  | *p= 0.584* | *p= 0.218* | *p= 0.168* |
| No | 1,158 (69.8) | 1.20 (1.02) | 0.69 (1.04) | 7.88 (0.12) |
| Yes | 501 (30.2) | 1.22 (1.03) | 0.75 (1.06) | 8.17 (0.18) |
| **Physical activity** |  | *p= 0.730* | *p= 0.308* | *p= 0.349* |
| Active | 1,234 (74.6) | 1.20 (1.02) | 0.72 (1.04) | 7.91 (0.11) |
| Inactive | 421 (25.4) | 1.21 (1.03) | 0.67 (1.06) | 8.12 (0.19) |
| **Obese** |  | *p< 0.001* | *p< 0.001* | *p< 0.001* |
| No | 1,424 (86.6) | 1.12 (1.02) | 0.62 (1.03) | 8.25 (0.10) |
| Yes | 221 (13.4) | 1.83 (1.05) | 1.57 (1.08) | 6.20(0.26) |

Interleukin-6 (IL-6) and C-Reactive Protein (CRP) analysis on logarithmic scale - results presented in exponential means.

Obesity - BMI > 2 z-score (15 years) or ≥ 30kg/m² (18 and 22 years)

Physical inactivity - < 300 min/week (15 and 18 years) or < 150 min/week (22 years)

Current smoker - > 6 days with cigarette consumption in the last month (15 years) or at least a cigarette /week in the last month (18 and 22 years).

Current alcohol intake - > 6 days with alcohol consumption in the last month (15 years) or harmful alcohol intake - AUDIT score ≥ 8 points (18 and 22 years).
