# Supplementary material for: Association of modifiable risk factors and IL-6, CRP, and adiponectin: Findings from the 1993 Birth Cohort, Southern Brazil
Source: PLoS One. 2019 May 9;14(5):e0216202. doi: 10.1371/journal.pone.0216202 (PMC6508856; doi:10.1371/journal.pone.0216202)
Supplement: S2 Table — The 1993 Pelotas Birth Cohort. (DOCX) [file pone.0216202.s003.docx]

S2 Table. Prevalence of risk factors according to age and mean (SE) levels of IL-6, CRP and adiponectin at 22 years, females. The 1993 Pelotas Birth Cohort.

|  | **Prevalence**  **N (%)** | **IL-6 (pg/mL)**  **Mean (SE)** | **CRP (mg/L)**  **Mean (SE)** | **Adiponectin (µg/mL)**  **Mean (SE)** |
| --- | --- | --- | --- | --- |
| **15 years** |  |  |  |  |
| **Current Smoker** |  | *p= 0.596* | *p=0.480* | *p= 0.026* |
| No | 1,659 (96.6) | 1.37 (1.02) | 1.60 (1.03) | 10.69 (0.12) |
| Yes | 59 (3.4) | 1.43 (1.08) | 1.35 (1.16) | 9.30 (0.61) |
| **Habitual alcohol intake** |  | *p= 0.867* | *p= 0.378* | *p= 0.451* |
| No | 1,628 (95.3) | 1.38 (1.02) | 1.61 (1.03) | 10.66 (0.12) |
| Yes | 81 (4.7) | 1.36 (1.07) | 1.41 (1.16) | 10.26 (0.52) |
| **Physical activity** |  | *p= 0.601* | *p= 0.404* | *p= 0.875* |
| Active | 600 (34.5) | 1.39 (1.03) | 1.54 (1.05) | 10.67 (0.19) |
| Inactive | 1,138 (65.5) | 1.37 (1.02) | 1.63 (1.04) | 10.64 (0.14) |
| **Obese** |  | p< 0.001 | p< 0.001 | p< 0.001 |
| No | 1,564 (93.0) | 1.32 (1.02) | 1.51 (1.03) | 10.78 (0.12) |
| Yes | 117 (7.0) | 2.31 (1.07) | 3.07 (1.13) | 8.58 (0.43) |
| **18 years** |  |  |  |  |
| **Current Smoker** |  | *p< 0.001* | *p= 0.763* | *p= 0.173* |
| No | 1,485 (88.0) | 1.34 (1.02) | 1.58 (1.03) | 10.70 (0.12) |
| Yes | 203 (12.0) | 1.64 (1.06) | 1.52 (1.10) | 10.22 (0.32) |
| **Harmful alcohol intake** |  | *p= 0.896* | *p= 0.496* | *p= 0.102* |
| No | 1,402 (83.1) | 1.37 (1.02) | 1.59 (1.04) | 10.73 (0.12) |
| Yes | 286 (16.9) | 1.38 (1.04) | 1.50 (1.08) | 10.23 (0.28) |
| **Physical activity** |  | *p= 0.870* | *p= 0.847* | *p= 0.286* |
| Active | 798 (47.3) | 1.37 (1.02) | 1.58 (1.05) | 10.77 (0.17) |
| Inactive | 888 (52.7) | 1.37 (1.02) | 1.57 (1.05) | 10.53 (0.16) |
| **Obese** |  | *p< 0.001* | *p< 0.001* | *p< 0.001* |
| No | 1,456 (89.3) | 1.28 (1.02) | 1.43 (1.03) | 10.89 (0.12) |
| Yes | 174 (10.7) | 2.43 (1.05) | 3.39 (1.09) | 8.73 (0.35) |
| **22 years** |  |  |  |  |
| **Current Smoker** |  | *p= 0.010* | *p= 0.208* | *p= 0.028* |
| No | 1,569 (86.4) | 1.35 (1.02) | 1.65 (1.03) | 10.82 (0.12) |
| Yes | 248 (13.6) | 1.53 (1.05) | 1.47 (1.09) | 10.11 (0.30) |
| **Harmful alcohol intake** |  | *p= 0.696* | *p= 0.773* | *p= 0.387* |
| No | 1,557 (85.7) | 1.38 (1.02) | 1.63 (1.03) | 10.68 (0.12) |
| Yes | 260 (14.3) | 1.35 (1.05) | 1.59 (1.08) | 10.95(0.29) |
| **Physical activity** |  | *p= 0.128* | *p= 0.665* | *p= 0.038* |
| Active | 1,054 (58.0) | 1.34 (1.02) | 1.60 (1.04) | 10.91 (0.15) |
| Inactive | 762 (42.0) | 1.42 (1.03) | 1.65 (1.05) | 10.45(0.17) |
| **Obese** |  | *p< 0.001* | *p< 0.001* | *p< 0.001* |
| No | 1,469 (81.3) | 1.20 (1.02) | 1.32 (1.03) | 11.28 (0.12) |
| Yes | 338 (18.7) | 2.53 (1.04) | 3.93 (1.06) | 8.29(0.25) |

Interleukin-6 (IL-6) and C-Reactive Protein (CRP) analysis on logarithmic scale - results presented in exponential means.

Obesity - BMI > 2 z-score (15 years) or ≥ 30kg/m² (18 and 22 years)

Physical inactivity - < 300 min/week (15 and 18 years) or < 150 min/week (22 years)

Current smoker - > 6 days with cigarette consumption in the last month (15 years) or at least a cigarette /week in the last month (18 and 22 years).

Current alcohol intake - > 6 days with alcohol consumption in the last month (15 years) or harmful alcohol intake - AUDIT score ≥ 8 points (18 and 22 year).
